# Stroke admission rates before, during and after the first phase of the COVID-19 pandemic

**DOI:** 10.1007/s10072-021-05039-y

**Published:** 2021-01-11

**Authors:** Espen Saxhaug Kristoffersen, Silje Holt Jahr, Kashif Waqar Faiz, Bente Thommessen, Ole Morten Rønning

**Affiliations:** 1 Department of Neurology, Akershus University Hospital, PO Box 1000, 1478 Lørenskog, Norway; 2 Department of General Practice, University of Oslo, Oslo, Norway; 3 Institute of Clinical Medicine, University of Oslo, Oslo, Norway

**Keywords:** Stroke, Pandemic, Health care planning, Emergency care, Stroke care pathways

## Abstract

**Background:**

There was a significant decrease in stroke admissions during the first phase of the COVID-19 pandemic. There are concerns that stroke patients have not sought medical attention and in the months after the lockdown suffer recurrent severe strokes. The aims of this study were to investigate how stroke admission rates and distributions of severity varied before, during and after the lockdown in a representative Norwegian hospital population.

**Methods:**

All patients discharged from Akershus University Hospital with a diagnosis of transient ischemic attack (TIA) or acute stroke from January to September 2020 were identified by hospital chart review.

**Results:**

We observed a transient decrease in weekly stroke admissions during lockdown from an average of 21.4 (SD 4.7) before to 15.0 (SD 4.2) during and 17.2 (SD 3.3) after (*p* < 0.011). The proportion of mild ischemic and haemorrhagic strokes was also lower during lockdown with 66% before, 57% during and 68% after (*p* = 0.011).

**Conclusion:**

The period of COVID-19 lockdown was associated with a temporary reduction in total admissions of strokes. In particular, there were fewer with TIA and mild stroke. Given the need to prevent the worsening of symptoms and risk of recurrence, it is necessary to emphasise the importance to seek medical care even in states of emergency.

## Introduction

Many stroke centres have reported that COVID-19 discourages patients from contacting the health care system with an increase in prehospital delay and decrease in stroke admissions as a result [1–14]. We have previously reported a decrease of almost 1/3 in stroke admissions in relation to the lockdown in Norway [6]. In our country as in other countries, the reduction in the total number of stroke admissions was assumed to be mainly due to a drop in admission of milder strokes [1–3, 6–8, 11–13]. It is feared that, given that the number of mild strokes in the population was unchanged during the pandemic, a large number of these patients did not seek medical attention. Patients with mild strokes are at risk of recurrent and more severe strokes without urgent treatment and initiation of secondary prevention [15, 16]. Consequently, people with mild strokes not admitted during the pandemic are now possibly at increased risk of future recurrent strokes, dependency or death. After the pandemic lockdown, there have been some anecdotal and mass media reports that the number of stroke patients seen in the emergency department has increased and that a larger proportion than usual have severe strokes. As far as we know, no rigorous studies on this subject describing the period after a national lockdown have been published.

The aim of this “Stroke during a pandemic (StrokePan) study” was to investigate how lockdown due to the COVID-19 pandemic affected the admission rates of transient ischemic attack (TIA) and acute stroke (ischemic and haemorrhagic), and distribution rates of mild, moderate and severe strokes in a representative Norwegian hospital population.

## Material and methods

### Setting

All patients initially admitted to and discharged from Akershus University Hospital with a diagnosis of TIA or acute stroke, both ischemic and haemorrhagic (International Classification of Diseases, version 10 codes G45.9, I61.x and I63.x), were identified by review of the electronic hospital diagnosis registry for patients admitted between January 3 and September 24, 2020. Akershus University Hospital is Norway’s largest emergency care hospital. Norwegian hospitals are almost exclusively publicly financed, and Norway has an all-covering national health insurance. Akershus University Hospital is the only hospital in the catchment area and is covering a population of 570.000, which is approximately 10% of Norway’s population. The hospital’s stroke unit is classified as a comprehensive stroke centre which includes endovascular therapy. The Norwegian national lockdown due to the COVID-19 pandemic was declared by March 12, 2020.

### Outcomes

To compare the weekly admission rates, the period from January 3 to March 12 was defined as *before lockdown* (weeks 1–10), the period from March 13 to April 30 was defined as *during lockdown* (weeks 11–17) and the period from May 1 to September 24 was defined as *after lockdown* (weeks 18–38). Stroke severity was defined by NIHSS on admission and categorised into mild (NIHSS ≤ 5), moderate (NIHSS 6–14) and severe (NIHSS ≥ 15). The proportion of different stroke severities includes both ischemic and haemorrhagic strokes but not TIAs.

### Statistics

For descriptive data, proportions, median, means and standard deviations (SDs) are given. Groups (before, during and after lockdown) were compared using the *t* test, the Wilcoxon (continuous data) test or the *χ*^2^ test (categorical data). Significance levels were set at *p* < 0.05, using two-sided test. Statistical analyses were performed using SPSS 26.00 (SPSS Inc., Chicago, IL, USA).

## Results

**Fig. 1:**
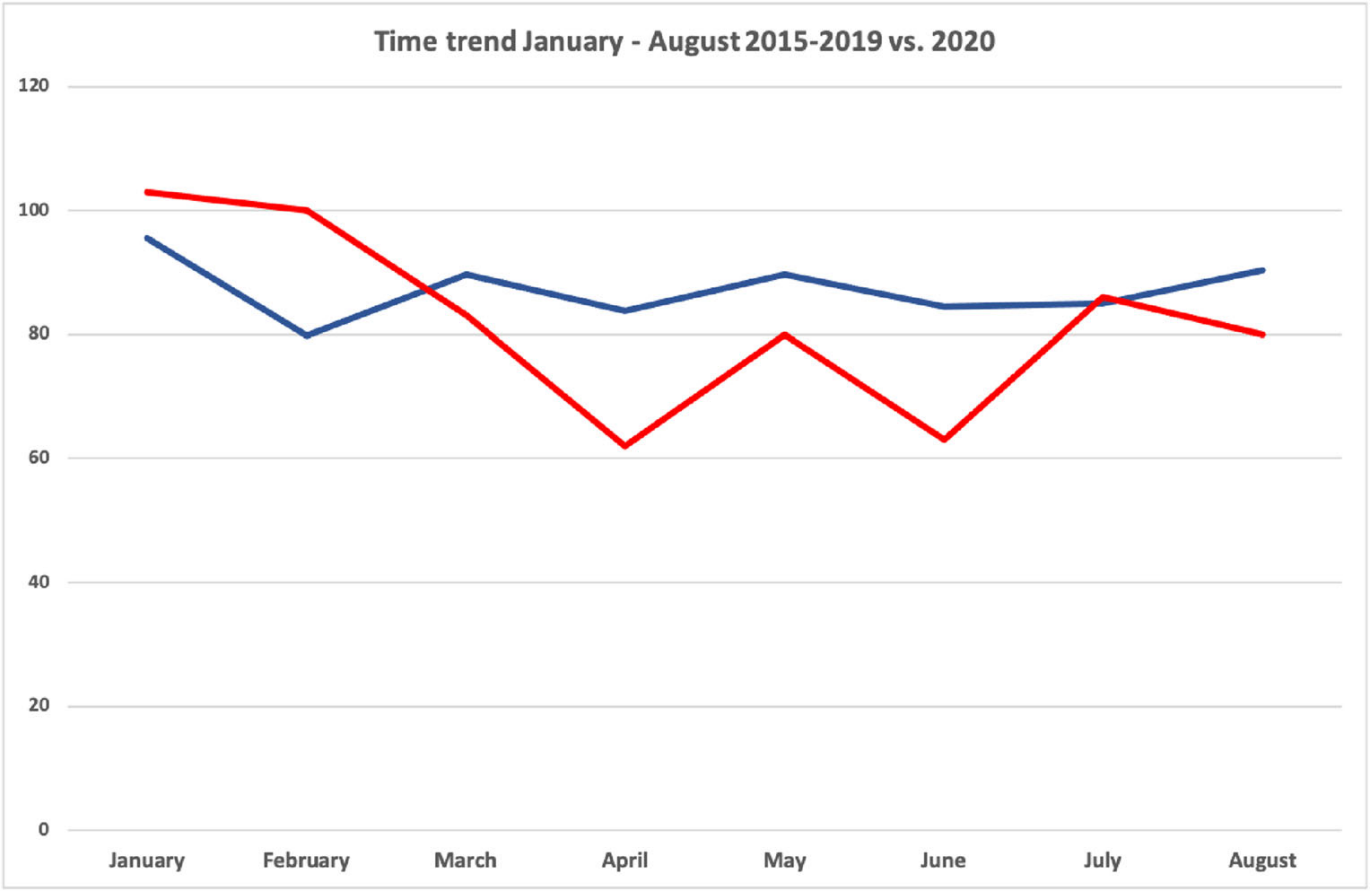
Month by month trend (January–August) for admissions due to acute stroke and transient ischemic attacks. Blue line: 2015–2019 (average). Red line: 2020

The overall trend for admissions due to stroke or TIAs to our hospital between January and August in the previous year 2015–2019 vs. 2020 is shown in Fig. 1. A detailed description of the type of stroke and admissions (*n* = 680) before (*n* = 214), during (*n* = 105) and after (*n* = 361) the national COVID-19 lockdown in 2020 is given in Table 1.

**Table 1:** Description of the total sample (*N* = 680)

	Before lockdown 3 January–12 March, *N* = 214	Lockdown 12 March–30 April, *N* = 105	After lockdown 1 May–24 September, *N* = 361	Total 3 January–24 September, *N* = 680
Age, mean years (SD)	73.4 (12.2)	75.0 (11.9)	72.7 (12.7)	73.3 (12.4)
Age ≥ 80 years, *n* (%)	71 (33)	45 (43)	115 (32)	231 (34)
Sex, women, *n* (%)	98 (46)	51 (49)	152 (42)	301 (44)
Living alone, *n* (%)	75 (35)	39 (37)	118 (33)	232 (33)
NIHSS at admission				
Median (IQR)	2 (6)	2 (6)	2 (5)	2 (6)
Mean (SD)	4.2 (6.2)	5.9 (8.6)	4.6 (7.2)	4.7 (7.2)
Stroke severity at admission for ischemic and haemorrhagic stroke, *n* (%)				
Mild (NIHSS ≤ 5)	111 (66)	49 (57)	190 (68)	328 (67)
Moderate (NIHSS > 5 or < 15)	46 (27)	22 (26)	49 (18)	104 (21)
Severe (NIHSS ≥ 15)	12 (7)	15 (17)	40 (14)	59 (12)
Reaching hospital within 4.5 h after onset, *n* (%)	99 (46)	41 (39)	148 (41)	288 (42)
Proportion of those with ischemic stroke reaching hospital within 4.5 h after onset, *n* (%)	65 (45)	23 (30)	73 (35)	161 (38)
Proportion of those with ischemic stroke that received thrombolysis, *n* (%)	36 (25)	14 (18)	37 (18)	87 (20)
Discharge diagnosis, *n* (%)				
Ischemic stroke	140 (65)	76 (72)	243 (67)	459 (68)
Haemorrhagic stroke	29 (14)	10 (9)	35 (10)	74 (11)
Transient ischemic attack	45 (21)	19 (18)	83 (23)	147 (22)
Hospital stay, mean days (SD)				
Total	5.8 (3.8)	5.1 (3.7)	5.3 (3.6)	5.4 (3.7)
Ischemic stroke	6.3 (3.7)	5.7 (3.9)	5.8 (3.3)	5.9 (3.5)
Haemorrhagic stroke	7.5 (4.7)	5.1 (2.8)	6.6 (6.2)	6.7 (5.3)
Transient ischemic attack	3.4 (2.1)	2.5 (1.5)	3.1 (1.8)	3.1 (1.9)
In-hospital mortality, *n* (%)	12 (6)	5 (5)	21 (6)	34 (6)

*NIHSS* National Institute of Health Stroke Scale

There were 21.4 (SD 4.7, range 29–14) weekly admissions before the lockdown, 15.0 (SD 4.2, range 21–8) during the lockdown and 17.2 (SD 3.3, range 22–12) after the lockdown (*t* test, *p* < 0.01 for before vs. during and before vs. after but not significant during vs. after).

The median weekly admissions before, during and after the lockdown were 21, 14 and 17 (*p* < 0.001).

The corresponding mean weekly admission figures (before, during and after lockdown) were 14.0, 10.9 and 11.6 for ischemic stroke (*p* < 0.05 for before vs. during and before vs. after); 2.9, 1.4 and 1.7 for haemorrhagic stroke (*p* < 0.05 for before vs. during and before vs. after); and 4.5, 2.7 and 4.0 for TIA (not significantly different).

**Fig. 2:**
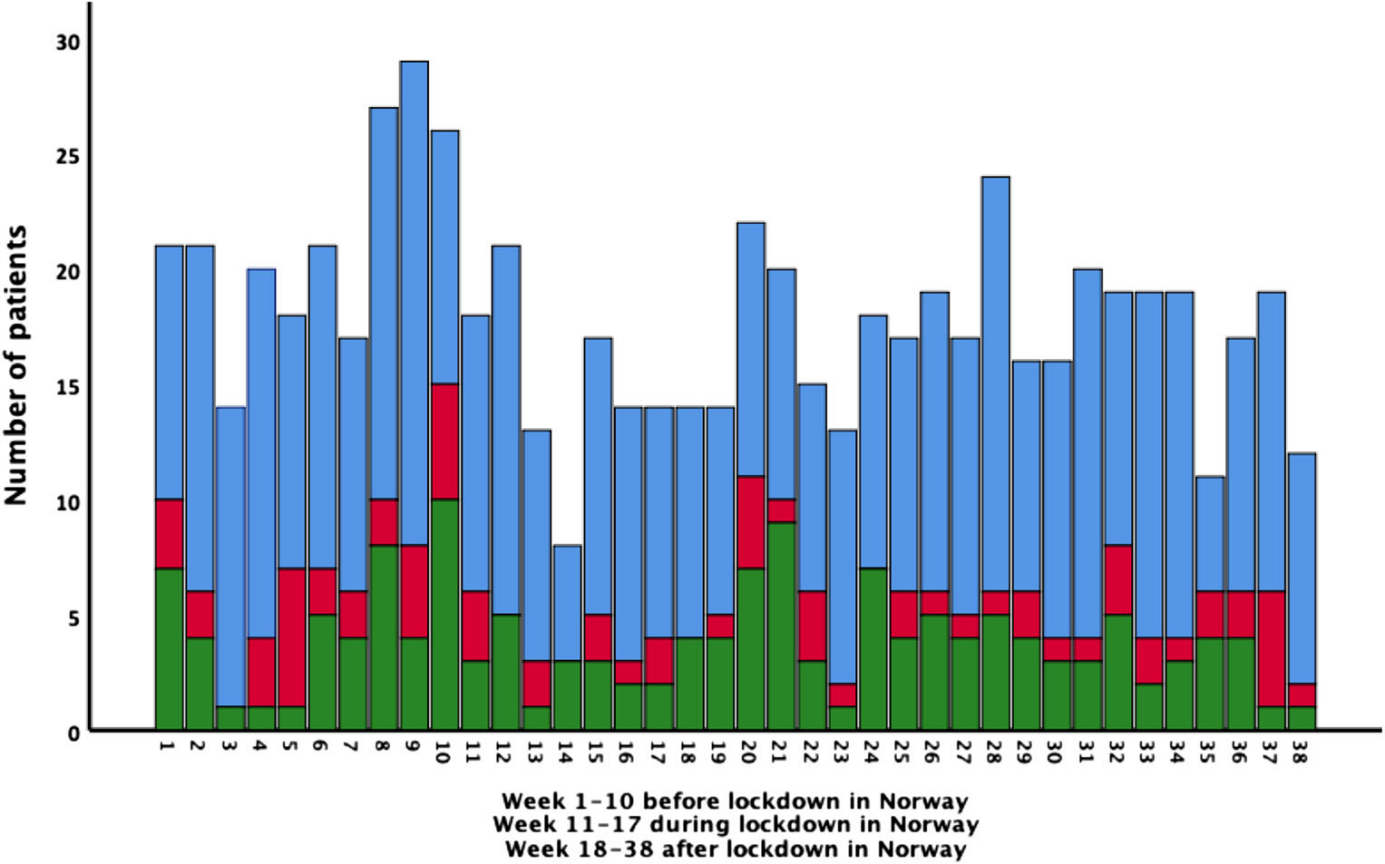
Weekly admission rates for transient ischemic attack (green), haemorrhagic stroke (red) and ischemic stroke (blue) before, during and after lockdown in 2020

Figure 2 shows the week by week distribution of admissions and types of stroke.

**Fig. 3:**
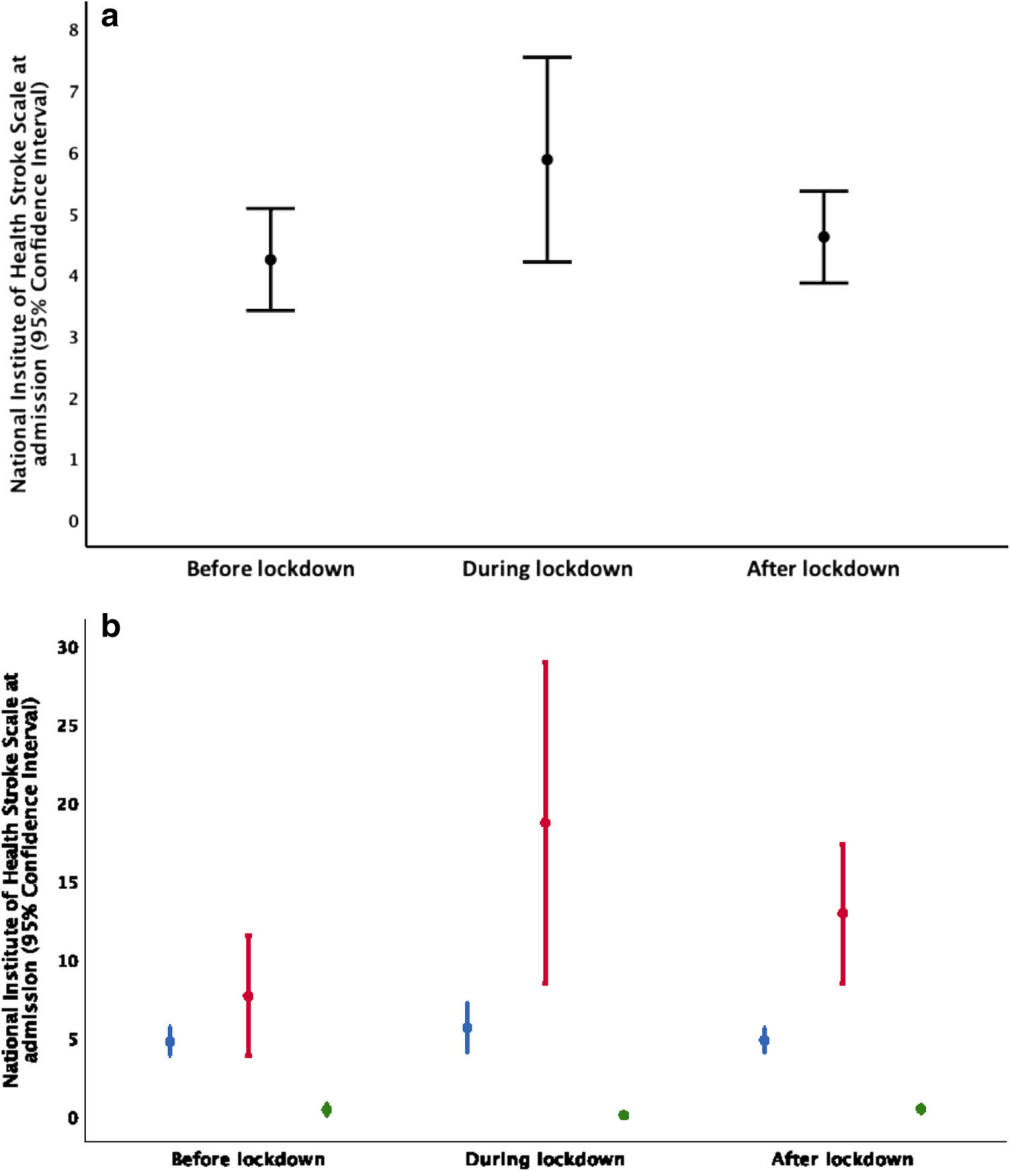
**a** The mean National Institute of Health Stroke Scale (NIHSS) (with 95% CI) before, during and after lockdown in 2020. **b** The mean National Institute of Health Stroke Scale (NIHSS) (with 95% CI) for transient ischemic attack (green), haemorrhagic stroke (red) and ischemic stroke (blue) before, during and after lockdown in 2020

Patients with strokes or TIAs had on average higher NIHSS at admission (Table 1) during the lockdown (5.9) as compared to before and after the lockdown (4.2 and 4.6; *p* < 0.05 comparing before vs. during lockdown), but not significantly different comparing before vs. after lockdown. Figure 3 a shows error bars (95% CI) of NIHSS for all stroke admissions and Fig. 3b NIHSS of the different subtypes of admissions for the periods before, during and after the lockdown. No patients admitted after 1 May reported a recent unadmitted TIA or stroke during the lockdown.

The stroke severity (mild, moderate and severe) also differed between the lockdown and the time periods before and after (Table 1; *χ*^2^ test, *p* = 0.011).

For patients with acute ischemic stroke (not including TIA), the mean NIHSS was 5.6, 4.7 and 4.8 during, before and after lockdown (not significant).

**Fig. 4:**
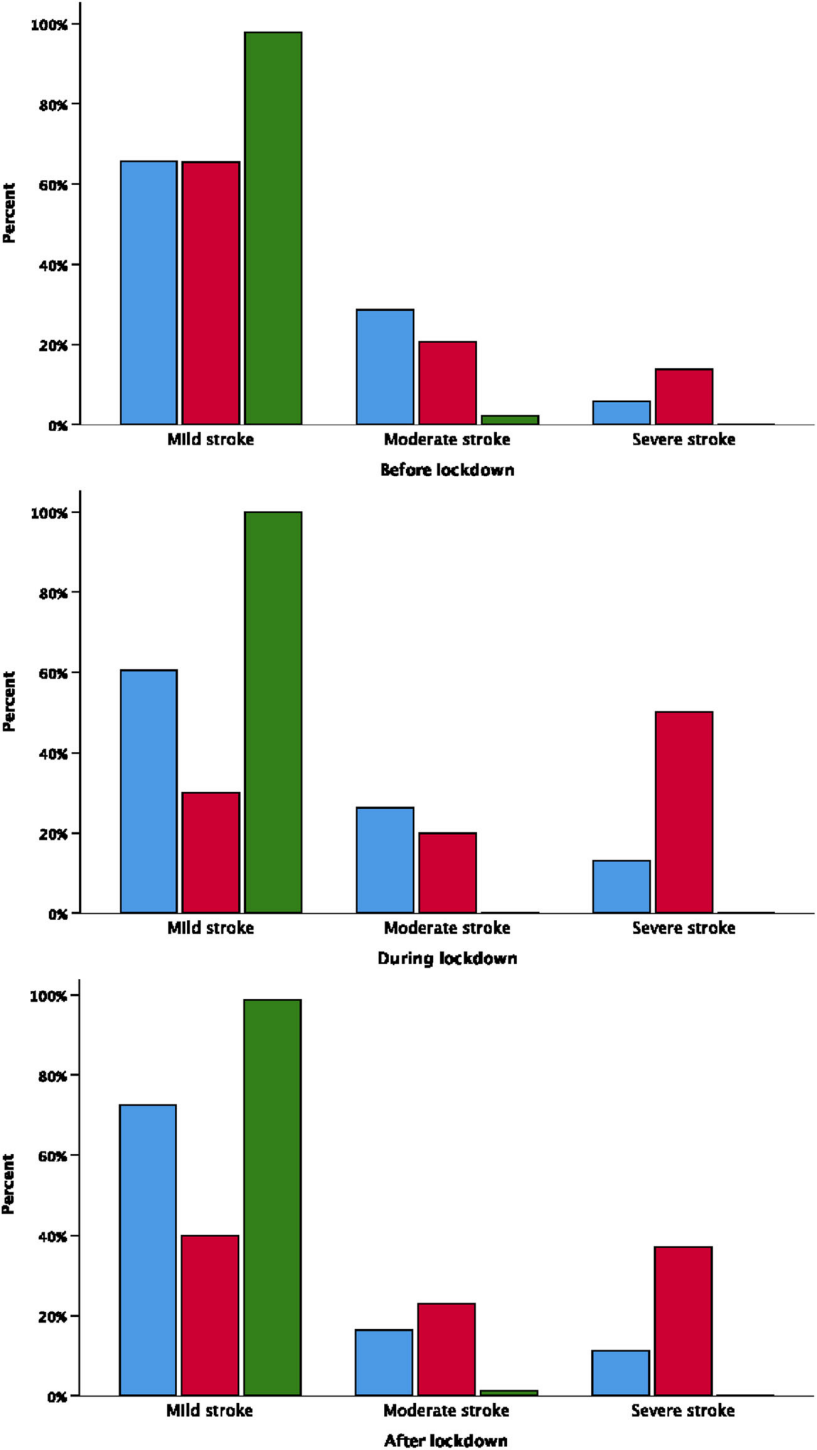
The proportion of stroke severities of transient ischemic attack (green), haemorrhagic stroke (red) and ischemic stroke before, during and after lockdown in 2020. Mild (NIHSS ≤ 5), moderate (NIHSS 6–14) and severe (NIHSS ≥ 15)

In total, 68% (*n* = 314), 22% (*n* = 100) and 10% (*n* = 45) had mild, moderate and severe ischemic strokes during these 9 months. The distribution of stroke severity for transient ischemic attack, ischemic stroke and haemorrhagic stroke before, during and after the lockdown is illustrated in Fig. 4. There was a significant difference in the distribution of mild, moderate and severe ischemic stroke severity in the three time periods with more severe strokes during (13%) the lockdown than before (6%) or after (11%) the lockdown (*χ*^2^ test, *p* = 0.018). No differences were found for haemorrhagic stroke. The proportion of those with ischemic stroke reaching hospital within 4.5 h (thrombolytic time window) was significantly different between the three time periods before (45%), during (30%) and after (36%) lockdown (*χ*^2^ test, *p* = 0.05). The proportion receiving thrombolytic therapy if admitted within 4.5 h did not differ before, during or after lockdown.

Overall, the length of hospital stay and in-hospital mortality did not differ between the different time periods or subtypes of admissions (Table 1).

## Discussion

This study confirms that there was a true decrease in hospital admissions for stroke and TIA and increased prehospital delay during the lockdown. Based on the monthly comparison (January–August) of the admissions in 2015–2020 (Fig. 1), we suggest that there is a reduction in admissions for stroke and TIAs during the lockdown. This is in accordance with published reports from across the world [1–3, 6–8, 11–13]. A decrease in stroke activation codes and stroke admissions has been reported, and since the reduction has been mainly described for mild or moderate strokes, patient-related factors and avoidance behaviour due to COVID-19 have been postulated [1–3, 6–8, 11–13]. This behaviour may have the unfortunate consequence that people with strokes not seeking help during the pandemic may be at increased risk of future recurrent strokes due to the lack of optimal secondary prevention [16].

Contrary to previous published studies, the present one includes stroke admissions as long as 5 months after the national lockdown [1–3, 6–8, 11–13]. Thus, this study shows the trend in stroke admissions in a longer period after a national lockdown. Comparison of the periods before and after the national lockdown with the actual lockdown period shows that the number of admissions after the lockdown in 2020 is comparable to that in the same periods in the preceding years (2015–2019) rather than the lockdown period.

The differences in admission rates between the lockdown and the period before and after lockdown are mainly driven by fewer mild strokes and especially fewer TIAs. However, there seems to be a lasting effect with fewer stroke admissions also after the lockdown. The reasons for this may be related to the same avoidance behaviour and concerns over contracting COVID-19 or a true decrease in stroke incidence. Whether social distancing, hygiene measures, less stressful lives, reduced infections, reduced air pollution or other unmeasured factors have contributed to a reduction in stroke incidence is not explored yet, but reduced admissions have also been reported for other cardiovascular diseases [17–19]. Norway was among the countries with the lowest number of COVID-19 patients and hence reduced capacities of emergency services due to the burden of these patients should probably not affect the admission rates. On the contrary, health authorities emphasised that suspected stroke victims should continue to seek medical attention and call the emergency medical services despite the lockdown. There are contradicting results regarding prehospital delay and the overall number and proportion of ischemic stroke patients receiving intravenous thrombolysis during the lockdown [1, 2, 5, 7, 8, 10–13] and reduced standard of care and delayed stroke care pathways with increased door-to-needle time have also been reported [1, 2, 5, 7, 8, 10–13]. The proportion receiving thrombolysis if they reached our hospital within the time window was similar for all three periods, indicating that the hospital’s acute stroke workflow and pathway were not affected to a degree harming patients [10, 20]. No changes in length of hospital stay or in-hospital mortality were found in our study. Comparison of changes in mortality, onset-to-door and door-to-needle time in different studies should be done with caution as other health care systems were flooded by COVID-19 patients with big implications for the emergency care pathways. In certain countries, hospitals were also changed to designated COVID-19 hospitals affecting the regular clinical workflow.

The main limitations to our study are that it is a single-centre retrospective study. However, Akershus University Hospital is Norway’s largest hospital covering approximately 10% of Norway’s population and is the only hospital in the catchment area, leaving an unselected population, which is also reasonably representative of the total Norwegian population.

Norway is a country with large geographical differences, and our findings may be different in more rural and remote areas with longer transfer to hospitals.

As far as we know, this is the first study to report on how the admissions of TIA and acute stroke (ischemic and haemorrhagic) differed before, during and after the lockdown due to the first phase of the COVID-19 pandemic.

## Conclusion

The period of lockdown due to the initial phase of the COVID-19 pandemic was associated with a temporary reduction in stroke admissions. In particular, there were fewer patients with TIA and mild stroke. Given the need to prevent the worsening of symptoms and risk of recurrence, it is necessary to emphasise the importance to seek medical attention even in times of declared emergency.

## Data Availability

The data that support the findings of this study are available on reasonable request from the corresponding author.
